# Epilepsy surgery without lipoma removal for temporal lobe epilepsy associated with lipoma in the Sylvian fissure

**DOI:** 10.1007/s00701-022-05330-7

**Published:** 2022-08-08

**Authors:** Kazuki Nomura, Hiroharu Suzuki, Yasushi Iimura, Takumi Mitsuhashi, Samantha Tamrakar, Tetsuya Ueda, Kazuki Nishioka, Keiko Fusegi, Mari Tada, Madoka Nakajima, Akiyoshi Kakita, Hidenori Sugano

**Affiliations:** 1 Department of Neurosurgery, Juntendo University, Tokyo, Japan; 2 Department of Neurosurgery, National Defense Medical College, Saitama, Japan; 3 Department of Pathology, Brain Research Institute, Niigata University, Niigata, Japan

**Keywords:** Temporal lobe epilepsy, Sylvian fissure, Intracranial lipoma, Epilepsy surgery, Intraoperative electrocorticography, Focal cortical dysplasia

## Abstract

Epileptic seizure is the common symptom associated with lipomas in the Sylvian fissure (Sylvian lipomas). Removal of these lipomas carries risks of hemorrhage and brain damage. We report a surgical strategy of not removing the lipoma in a case of intractable temporal lobe epilepsy associated with Sylvian lipoma. We performed anterior temporal lobectomy with preservation of the pia mater of the Sylvian fissure and achieved seizure freedom. Focal cortical dysplasia type 1 of the epileptic neocortex adjacent to the Sylvian lipoma was pathologically diagnosed. We recommend our surgical procedure in similar cases to avoid complications and achieve adequate seizure control.

## Introduction

Intracranial lipomas are congenital malformations that occur in 0.46–1% of intracranial tumors [3]. These lipomas are primarily localized to the interhemispheric area, followed by localization to the dorsal brain stem [19], with the majority of them not causing life-threatening symptoms [16]. Removal of the lipomas may result in postoperative hemorrhage and brain parenchymal damage, owing to the strong attachment to the surrounding tissue [5, 16]. Therefore, no consensus exists on surgical indication for the removal of intracranial lipomas in most cases.

Lipomas in the Sylvian fissure (Sylvian lipomas) are extremely rare, with incidences of 3.4% and 5% in case series of intracranial lipomas by Maiuri et al. [11] and Truwit et al. [19], respectively. Sylvian lipomas are clinically associated with epileptic seizures, particularly focal impaired awareness seizures (FIAS) [5]. In some cases, the seizures are refractory to antiepileptic drugs, requiring the consideration of surgical treatment for the epilepsy [1]. The accurate identification of the epileptic focus and prevention of complications are the two most important factors for the safe and effective surgical treatment of patients with temporal lobe epilepsy (TLE) due to Sylvian lipoma. Here, we attempted a strategy of identifying the epileptic focus using intraoperative electrocorticography (ioECoG) and preventing complications by not removing the lipoma during the removal of the epileptic region.

## Case presentation

**Fig. 1:**
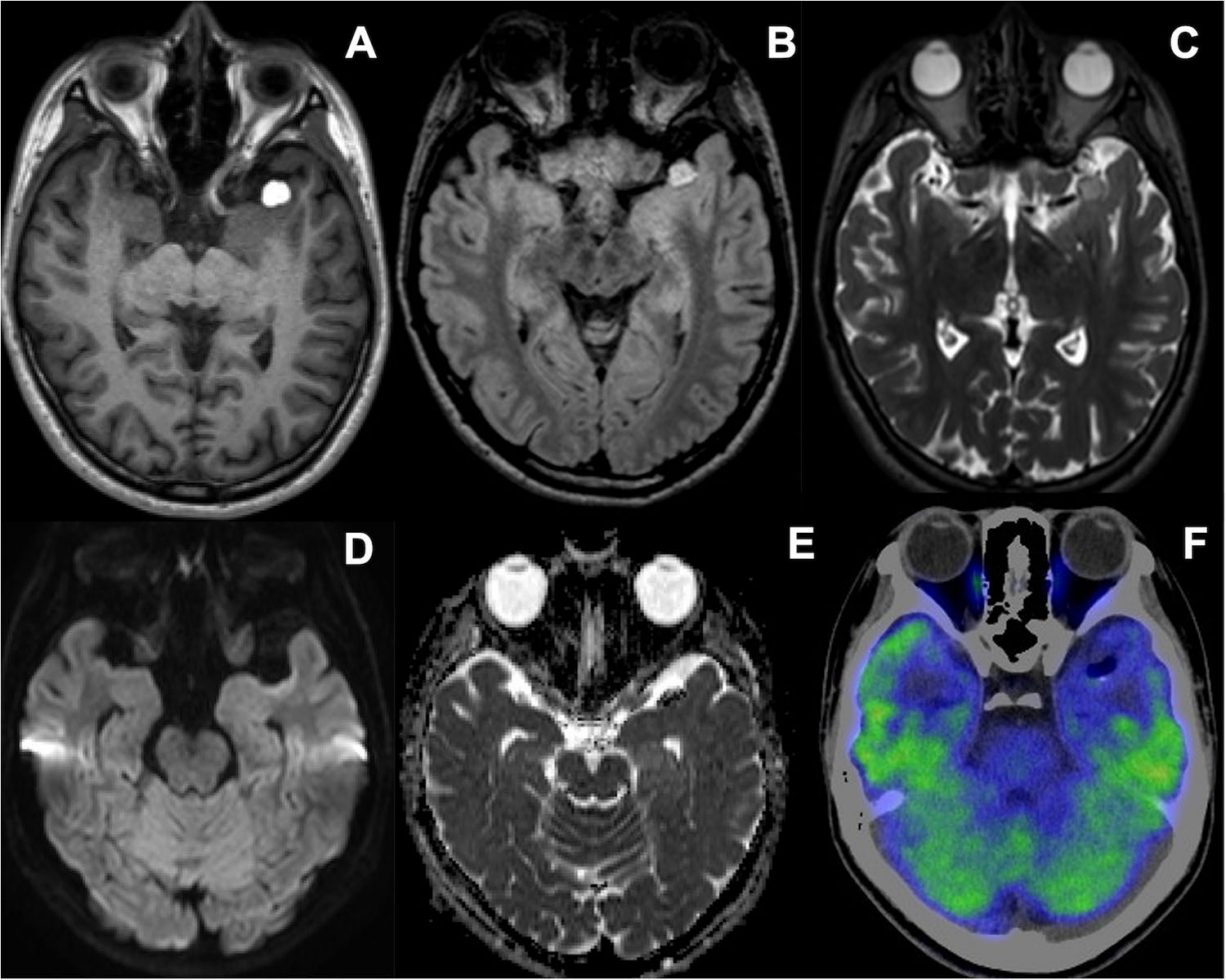
The Sylvian lipoma shows a markedly high signal intensity on axial T1-weighted imaging (**A**) and fluid-attenuated inversion recovery imaging (**B**), along with a slightly high signal intensity on axial T2-weighted imaging (**C**) on preoperative magnetic resonance imaging. The Sylvian lipoma shows signal supression on axial diffusion-weighted imaging (**D**) and the corresponding apparent diffusion coefficient map (**E**). The anterior temporal cortex adjacent to the Sylvian lipoma shows hypometabolism on fluorodeoxyglucose positron emission tomography/computed tomography (**F**)

A 19-year-old woman had FIAS presented with an aura of olfactory hallucinations, followed by behavior arrest with prominent oroalimentary automatism. She had no history of having focal to bilateral tonic–clonic seizures. Her FIAS was not controlled for more than 2 years even with 1000 mg of levetiracetam (LEV), with a frequency of one seizure per week. Her language dominancy was left (right-handed) on The Edinburgh Handedness Inventory Test [13]. Wechsler Memory Scale-Revised (WMS-R) demonstrated no memory decline (verbal memory 94, visual memory 103, general memory 96, attention/concentration 105, delayed recall 96) before surgery. Magnetic resonance imaging (MRI) showed a mass lesion in the left Sylvian fissure with homogeneous hyperintensity on both T1-weighted and T2-weighted images but no diffusion restriction on diffusion-weighted images/apparent diffusion coefficient maps (Fig. 1). Fluid-attenuated inversion recovery images demonstrated normal signal in the hippocampus. Fluorodeoxyglucose positron emission tomography revealed severe focal hypometabolism in the left anterior temporal lobe. Preoperative long-term scalp video-EEG monitoring (EEG-1200, Nihon Kohden, Tokyo, Japan) using the 10–20 international system for 3 days did not show ictal discharges but detected interictal epileptiform discharges (IEDs) at T1 and F7 electrodes with reproducibility. We concluded that the patient’s epilepsy was medically intractable and a surgical intervention for the TLE was needed. We applied the surgical strategy of identifying the epileptic focus using ioECoG and resecting it without removal of the Sylvian lipoma.

**Fig. 2:**
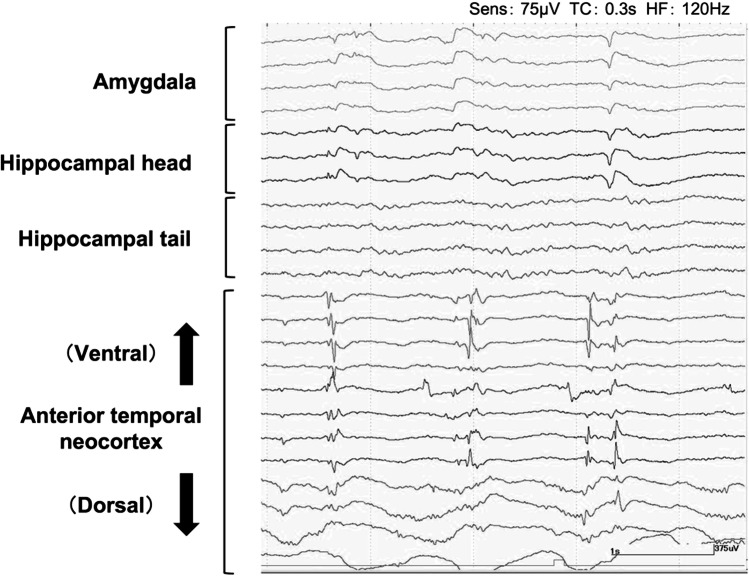
Intraoperative electrocorticography using an epidural electrode as reference shows the high-amplitude spikes on the ventral side of the anterior temporal lobe

**Fig. 3:**
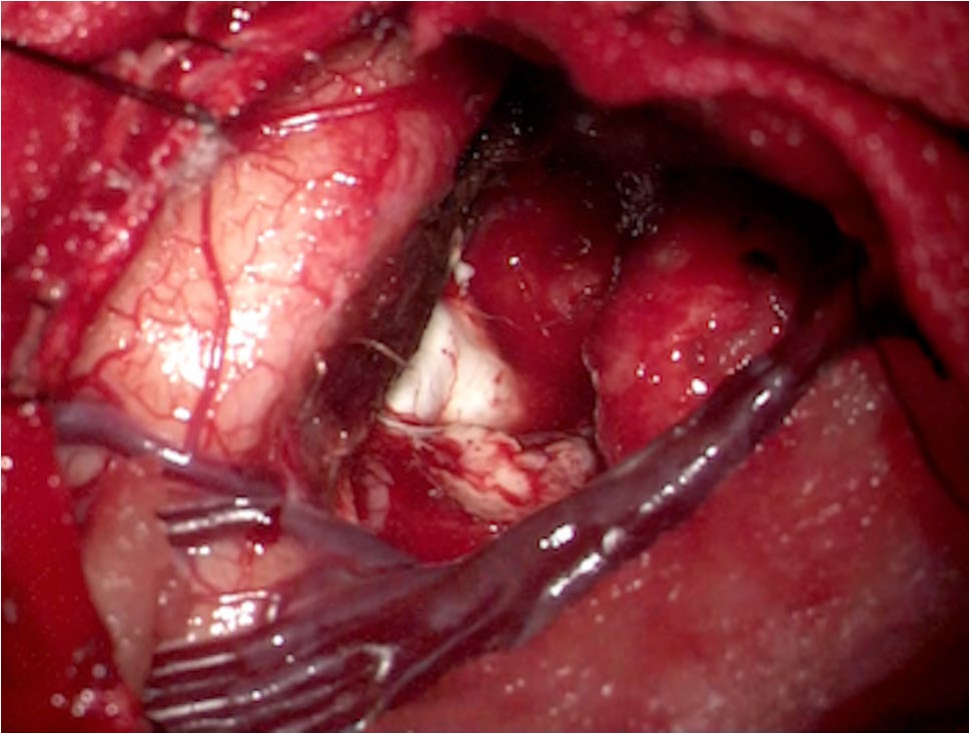
The intraoperative image after the anterior temporal lobectomy shows the remaining temporal lobe, hippocampus, and Sylvian lipoma covered with the pia mater of the Sylvian fissure

**Fig. 4:**
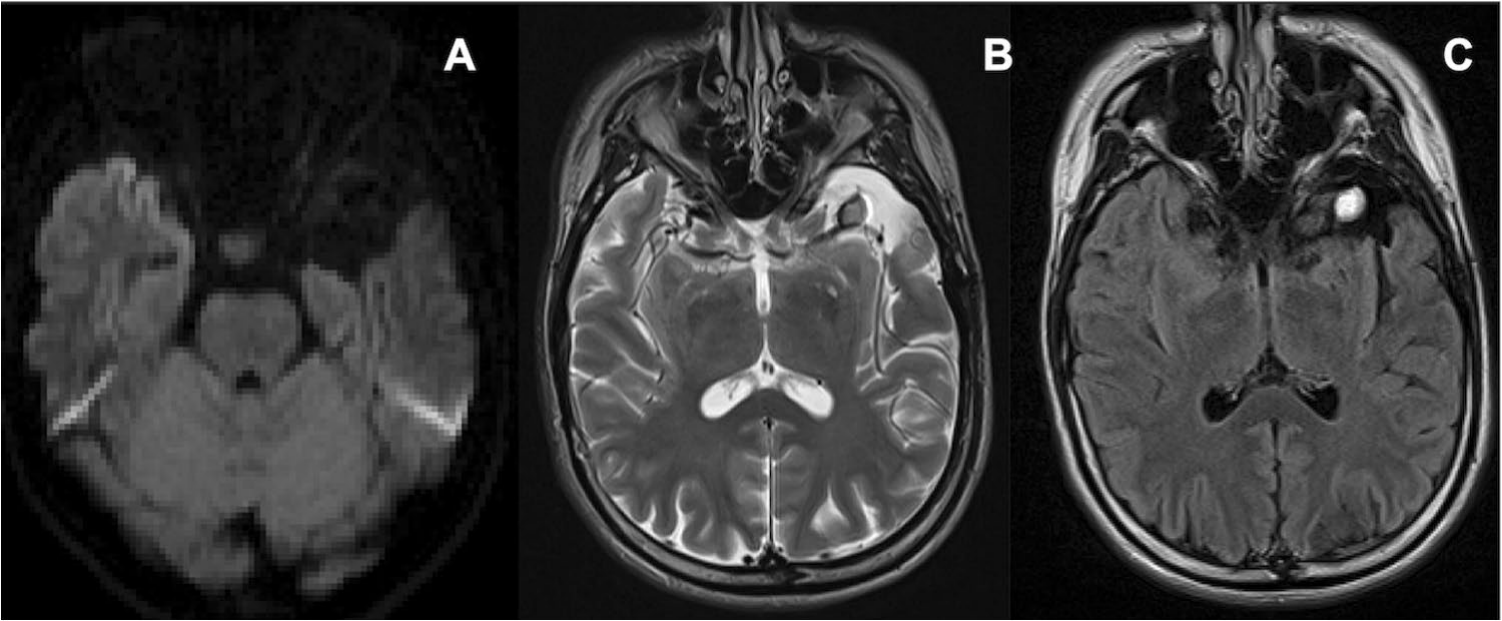
After the anterior temporal lobectomy, the Sylvian lipoma is shown isolated from the temporal lobe on axial diffusion-weighted (**A**), T2-weighted (**B**), and fluid-attenuated inversion recovery imaging (**C**)

After the pterional craniotomy, subpial dissection was initiated along the upper border of the superior temporal gyrus to protect the pia mater of the Sylvian fissure and approached to the inferior horn of the lateral ventricle. After obtaining an adequate space to observe the amygdala and the hippocampus, a 10-min ioECoG was recorded on the surface of the amygdala, hippocampus, and anterior temporal lateral cortex adjacent to the Sylvian lipoma using platinum electrodes (Unique Medical, Tokyo, Japan). The ioECoG showed IEDs at the strip electrodes located on the anterior lateral temporal lobe (Fig. 2). Therefore, we performed the anterior temporal lobectomy (ATL) 3 cm from the temporal tip without removing the Sylvian lipoma (Fig. 3). Postoperative MRI demonstrated the preserved Sylvian lipoma and branches of the middle cerebral artery within the Sylvian fissure (Fig. 4). Histopathological examination of the neocortical specimen of the anterior temporal lobe revealed neuronal cytoarchitectural abnormalities in the cortex. Thus, focal cortical dysplasia (FCD) type 1 of the anterior temporal neocortex was diagnosed.

At 2 years postoperatively, she never had seizures with 1000 mg of LEV without any neurological sequelae. Furthermore, follow-up EEG found no IEDs, while WMS-R 6 months after surgery indicated no memory decline (verbal memory 105, visual memory 114, general memory 99, attention/concentration 103, delayed recall 108).

## Discussion

### Detection of epileptic focus using ioECoG in TLE due to Sylvian lipoma

Based on the ioECoG findings, we concluded that the neocortex adjacent to the Sylvian lipoma was the epileptic focus. Consequently, ATL with the preservation of the Sylvian lipoma was performed that resulted in seizure freedom.

Temporal lobe tumors, particularly low-grade gliomas and glioneuronal tumors, are common causes of intractable TLE and consequently have been described as long-term epilepsy-associated tumors (LEATs) [20]. One of the surgical strategies for TLE with LEATs includes gross total tumor resection alone for seizure control; however, an adequate surgical strategy for TLE with LEATs has not been fully established [12]. Jooma et al. reported that the resection of the mesial temporal structures is an important factor for surgical success in refractory TLE because both the lesion and hippocampus could have increased epileptogenicity [8]. In another study, Sugano et al. evaluated the efficacy of the additional removal of electrically positive foci using ioECoG in cases of TLE with LEATs, and they concluded that ioECoG monitoring was valuable for detecting epileptic foci of the mesial temporal structures [18]. However, the contribution of the peritumoral tissue to the epileptogenicity also remains unclear [8], although Pelliccia et al. and Giulioni et al. reported a relationship between epilepsy and tumor-associated FCD [6, 14]. Nowak et al. found that extended temporal lobe resection provided for optimal resection of the epileptogenic tissue by removing the invisible foci of highly epileptogenic cortical dysplasia adjacent to the tumors [12].

Sylvian lipomas are extramedullary lesions; thus, an epileptic mechanism of LEATs may not be applicable in TLEs with Sylvian lipoma. Moreover, in this case, the epilepsy may have been caused by invisible foci of cortical dysplasia as MRI-negative TLE with no relation to the lipoma [15]. However, a lipoma is thought to be an aberrant tissue of the meninx primitiva, the mesenchymal precursor of the leptomeninges, formed during the development of the subarachnoid cisterns. Thus, the formation of a cerebral lipoma might be part of a complex malformation that also involves vascular abnormalities and abnormal cortical development within its vicinity [5, 9, 16]. Kakita et al. reported a polymicrogyric appearance in the cerebral cortex underlying the lipoma and demonstrated that focal disturbances in cortical development occur in association with the development of lipomas [9]. The abnormalities of the cortical laminae could be an etiology of epilepsy, which is supported by the histopathological diagnosis in our case. The epileptic focus in patients with Sylvian lipoma could be in the mesial temporal cortex or the variety of associated with neocortical abnormalities [5, 9, 16] In surgical cases of TLE due to Sylvian lipoma, we propose the strategy of detecting the epileptic focus within the mesial temporal structures and surrounding neocortical tissues using ioECoG.

### Surgical technique for preventing complication and achieving satisfactory seizure outcome

In cases where the cortex underlying the lipoma is evaluated to be the epileptic focus, the decision to remove the lipoma remains controversial. Surgical excision of intracranial lipomas is associated with serious hemorrhagic complications and the unfavorable seizure outcome [2, 4, 7, 11]. The tight adherence of the lipoma to the adjacent cortical and intervening vascular structures is a crucial factor that makes surgical excision difficult [5].

Dermoid cysts in the temporal lobe are another type of extramedullary benign tumor that can cause intractable TLE [17]. Li et al. reported a case of a dermoid cyst in the Sylvian fissure and discussed that the lipid content in the dermoid cyst could cause chemical irritation or meningitis and consequent epileptic discharge [10]. Hence, cyst rupture may be a risk factor for new-onset seizures in patients with Sylvian fissure dermoid cyst. We considered this possibility in our lipoma case as well. Thus, if we damaged the fibrous capsule surrounding the lipoma, secondary epileptogenic activity due to the lipid content might have developed in our case. In surgical cases of TLE due to Sylvian lipoma, we propose the surgical strategy of not removing the lipoma for avoiding irritation or meningitis caused by its lipid content and hemorrhagic complications associated with its strong adhesion to the surrounding tissue. Subpial dissection could be an appropriate technique to preserve the pia mater of the Sylvian fissure, thereby preventing damage to the Sylvian lipoma.

## Conclusion

We reported a surgical strategy using ioECoG to identify the epileptic focus in the TLE due to Sylvian lipoma for achieving favorable seizure outcome. Furthermore, with regard to th resection of the epileptic focus, ATL with subpial dissection to protect the Sylvian fissure and isolate the lipoma might be a reasonable surgical technique considering the lipoma formation and presence of FCD type 1 changes in the adjacent cortex.

## Abbreviations

ATL: Anterior temporal lobectomy
EEG: Electroencephalography
FCD: Focal cortical dysplasia
FIAS: Focal impaired awareness seizures
IEDs: Interictal epileptiform discharges
ioECoG: Intraoperative electrocorticography
LEATs: Long-term epilepsy-associated tumors
LEV: Levetiracetam
MRI: Magnetic resonance imaging
TLE: Temporal lobe epilepsy
WMS-R: Wechsler Memory Scale-Revised
